# Investigations into the elimination profiles and metabolite ratios of micro-dosed selective androgen receptor modulator LGD-4033 for doping control purposes

**DOI:** 10.1007/s00216-021-03740-7

**Published:** 2021-11-04

**Authors:** Felicitas Wagener, Sven Guddat, Christian Görgens, Yiannis S. Angelis, Michael Petrou, Andreas Lagojda, Dirk Kühne, Mario Thevis

**Affiliations:** 1 Center for Preventive Doping Research/Institute of Biochemistry, German Sport University Cologne, Am Sportpark Müngersdorf 6, 50933 Cologne, Germany; 2 European Monitoring Center for Emerging Doping Agents (EuMoCEDA), Cologne/Bonn, Germany; 3 Doping Control Laboratory of Athens, Institute of Biosciences & Applications, National Center for Scientific Research “Demokritos”, Neratziotissis & Amaryssias Artemidos Str, 15123 Athens, Greece; 4 Cyprus Anti-Doping Authority, Makarion Athletic Centre Avenue, Engomi, CY 2400 Nicosia, Cyprus; 5 BayerCropScience AG, Alfred-Nobel-Str. 50, 40789 Monheim, Germany

**Keywords:** Sport, Doping, SARMs, LGD-4033, Ligandrol, Metabolism

## Abstract

LGD-4033 (ligandrol) is a selective androgen receptor modulator (SARM), which is prohibited in sports by the World Anti-Doping Agency (WADA) and led to 62 adverse analytical findings (AAFs) in 2019. But not only deliberate doping with LGD-4033 constitutes a problem. In the past years, some AAFs that concerned SARMs can be attributed to contaminated dietary supplements (DS). Thus, the urgency to develop methods to differentiate between inadvertent doping and abuse of SARMs to benefit from the performance-enhancing effect of the compound in sports is growing. To gain a better understanding of the metabolism and excretion patterns of LGD-4033, human micro-dose excretion studies at 1, 10, and 50 µg LGD-4033 were conducted. Collected urine samples were prepared for analysis using enzymatic hydrolysis followed by solid-phase extraction and analyzed via LC-HRMS/MS. Including isomers, a total of 15 phase I metabolites were detected in the urine samples. The LC-HRMS/MS method was validated for qualitative detection of LGD-4033, allowing for a limit of detection (LOD) of 8 pg/mL. The metabolite M1, representing the epimer of LGD-4033, was synthesized and the structure elucidated by NMR spectroscopy. As the M1/LGD-4033 ratio changes over time, the ratio and the approximate LGD-4033 concentration can contribute to estimating the time point of drug intake and dose of LGD-4033 in doping control urine samples, which is particularly relevant in anti-doping result management.

**Graphical abstract:**

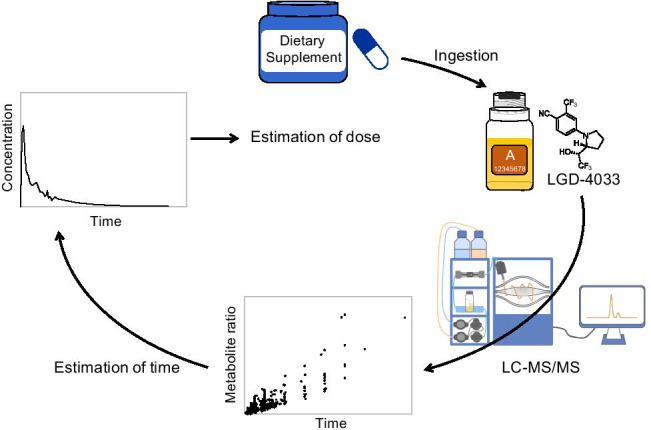

## Introduction

Selective androgen receptor modulators (SARMs) are a novel class of tissue-selective androgen receptor (AR) ligands, which have been in development since the end of the twentieth century [1]. SARMs have been developed for indications such as male contraception, treatment of muscle wasting, and cancer. SARMs show agonistic activity towards AR in muscle and bone while showing reduced or no activity towards AR in other tissues such as prostate and seminal vesicles [2, 3]. Due to their tissue selectivity, SARMs demonstrate reduced side effects such as hypogonadism, aggression, and gynecomastia compared to the use of anabolic androgenic steroids (AAS) [4]. Because of their potential to be abused in sporting competitions, SARMs are explicitly prohibited by the World Anti-Doping Agency (WADA) since 2008 [5].

LGD-4033 (ligrandol) is a non-steroidal SARM that provides significant increase in lean body mass at doses as low as 1 mg per day for 21 days and was shown to be well tolerated at this dose [6]. In higher doses, it has been linked to liver damage [7, 8]. Despite the fact that no SARM has received clinical approval yet, the number of adverse analytical findings (AAFs) with SARMs in sports drug testing has increased continuously in the past 5 years. In 2019, 62 AAFs with LGD-4033 were reported by the WADA, the highest number reported so far. Additionally to deliberate doping with LGD-4033, contaminated dietary supplements (DS) pose a risk of unintentional doping. As the analytical capabilities keep improving, even urinary drug and drug metabolite concentrations resulting from administration scenarios far below the expected effective dose can be detected. Although there are resources available to athletes to receive information about which DS might be of “low risk,” the possibility of ingesting prohibited substances by DS remains [9–11]. Due to the principle of strict liability as defined in the World Anti-Doping Code, athletes might face situations of an anti-doping rule violation (ADRV) [12] whenever a prohibited substance is detected in their doping control specimens. The resulting period of ineligibility may only be reduced if the athlete can prove beyond a reasonable doubt that the AAF was a result of a contaminated DS or otherwise not administered intentionally [13]. Since this represents a challenging task for most athletes, the development of analytical methods giving insight into dose and time post-administration might provide additional evidence for best-possible result management, similar to micro-dose elimination studies conducted earlier with the SARM ostarine [9, 14].

Several in vitro studies regarding the metabolism of LGD-4033 were conducted in the context of doping controls [15, 16]. Sobolevsky et al. published the results of the first human excretion study in 2015, in which they discovered mono- and bis-hydroxylated metabolites, as well as hydroxylated and ring-cleaved metabolites [17]. Fragkaki et al. conducted a human excretion study in which 11 metabolites (including isomers) were detected, with a maximum detection time of 20.5 days for the bis-hydroxylated metabolite [18]. Two studies investigating the metabolism and excretion behavior in horses were also published [19, 20].

Using liquid chromatography-tandem mass spectrometry (LC–MS/MS), Cox et al. were first to postulate the in vivo epimerization of LGD-4033 by observing a peak with the same exact mass as LGD-4033 and a shift to a longer retention time [21]. Fragkaki et al. documented during their excretion study with 10 mg LGD-4033 that the second peak got progressively more intense the more time had passed since the ingestion of LGD-4033 [18]. The exclusive concentration of LGD-4033 is not sufficient to differentiate between a high dose of LGD-4033 taken multiple days before the urine sample was collected and a small dose taken the day of the sample collection. But as the metabolite ratio shifts over time, this ratio is a promising target to gain additional insight into the time line of LGD-4033 ingestion.

## Methods

### Chemicals and materials

LGD-4033 reference material was obtained from SelleckChem (Houston, TX, USA). As internal standard (ISTD), the arylpropionamide-derived SARM S-24 was used and synthesized in-house as described previously [22]. Acetonitrile (ACN) and disodium hydrogen phosphate monohydrate (Na_2_HPO_4_∙H_2_O) were obtained from VWR chemicals (Radnor, PA, USA). Dichloromethane (DCM), sodium hydroxide (NaOH), sodium borohydride (NaBH_4_), and Dess-Martin periodinane were purchased from Sigma-Aldrich (St. Louis, MO, USA). *Tert*-butyl methyl ether (*t*BME) was obtained from PanReac AppliChem (Darmstadt, Germany). Ethanol (EtOH), ammonium acetate (NH_4_Ac), acetic acid (AcH), and sodium dihydrogen phosphate (NaH_2_PO_4_) were obtained from Merck (Darmstadt, Germany). Formic acid (FA) was obtained from Biosolve (Valkenswaard, Netherlands). Methanol (MeOH) was purchased from J.T.Baker (Phillipsburg, NJ, USA). Ultrapure water was received from a Barnstead GenPure xCAD Plus from Thermo Scientific (Bremen, Germany). β-Glucuronidase (*Escherichia coli*) was purchased from Roche Diagnostics (Mannheim, Germany).

### Synthesis of M1

The metabolite M1, which corresponds to the epimer of LGD-4033, was synthesized by solving 1 mg of LGD-4033 in 20 µL of DCM, adding a suspension of 5 mg of Dess-Martin periodinane in 40 µL of DCM, and stirring at RT. After 30 min, 200 µL of *t*BME and 200 µL of 2 M aqueous NaOH were added. The organic phase was separated and washed with 200 µL of 2 M aqueous NaOH. The organic phase was dried and reconstituted in 100 µL of 80% EtOH, and 5 mg of NaBH_4_ was added. The mixture was stirred at RT for 48 h, after which it was quenched with water and extracted with *t*BME, and the solvent was evaporated. The crude product was separated on a preparative LC system consisting of a HPLC Pump 1800, a Smartline Autosampler 3950, a Smartline UV Detector 2600 (all from Knauer, Berlin, Germany), and a Foxy R1 fraction collector (Teledyne Isco, Lincoln, NE, USA). A Eurospher II 100–10 C18 column (250 × 4 mm, Knauer, Berlin, Germany) was used with 0.1% FA as solvent A and ACN as solvent B. The separation was conducted with a flow rate of 1 mL/min and a linear gradient with 55% B as starting conditions, which were held for 4 min, increased to 65% B during 8 min, and increased to 100% B in 0.5 min and held for 2 min. The column was re-equilibrated at 55% B for 3.4 min. The injection volume varied between 10 and 100 µL. The UV detector was used at a wavelength of 305 nm. The target structure was confirmed via ^1^H-NMR spectroscopy.

### Elimination studies

A stock solution of 1 mg/mL of LGD-4033 in ethanol was prepared and the corresponding amount for a total of 1, 10, or 50 µg was added to 120 mL of drinking yoghurt. Five healthy male volunteers ingested the spiked yoghurt and collected urine samples. One blank urine sample was collected before the ingestion of LGD-4033. For the single-dose application study, a sample of every urine was collected during the first 48 h. After that, one sample was collected every 24 h until no analytes related to LGD-4033 were traceable. For the multi-dose application study, a spiked yoghurt was consumed on five consecutive days every morning. A sample of every urine was collected during the first 48 h, four samples per day for another 72 h, and subsequently one sample every 24 h until urine samples tested negative for all target analytes. Informed written consent of all volunteers and approval from the responsible ethical committee were obtained before the start of the study. The urine samples were stored at + 4 °C for a maximum time of 7 days before being transferred into long-term storage at − 20 °C. Before analysis, the samples were allowed to thaw at RT.

### Sample preparation

To 2 mL of urine, 10 µL of ISTD (S-24, 10 ng/mL in ACN), 1 mL of sodium phosphate buffer (0.8 M, pH 7.0), and 50 µL of β-glucuronidase were added before incubating at 50 °C for 1 h. After allowing to cool to RT, a SPE was conducted. For this, Chromabond HLB 3 mL/60 mg cartridges (Macherey–Nagel, Düren, Germany) were equilibrated with 3 mL of MeOH and H_2_O before loading with the entire hydrolyzed sample, washing with 3 mL of water, and eluting with 1 mL of MeOH. The MeOH was evaporated to dryness and the sample reconstituted in 100 µL of a mixture of ACN/NH_4_Ac buffer (5 mM, pH 3.5) 1:3, v/v. For all volume measurements, Research plus pipettes from Eppendorf (Hamburg, Germany) were used with a maximum deviation of ± 1%.

### LC–MS/MS analysis

The LC–MS/MS analysis was performed using a Vanquish HPLC system coupled to an Orbitrap Exploris 480, both from Thermo Fisher (Bremen, Germany). The chromatography was carried out using a EC 4/2 Nucleodur C-18 Pyramid 3 μm (4 × 2 mm) pre-column and an EC 50/2 Nucleodur C-18 Pyramid 1.8 μm (50 × 2 mm) analytical column (Macherey–Nagel, Düren, Germany). Five nanomole NH_4_Ac buffer containing 0.1% acetic acid (pH 3.5) was used as solvent A, and ACN was used as solvent B. The employed linear LC gradient had a total run time of 16.5 min and was set as follows: starting conditions 25% B and flow rate of 200 µL/min, increasing in 5 min to 40% B and in further 6 min to 100% B. The 100% B was maintained for 1 min at 200 µL/min and 1 min at 350 µL/min followed by re-equilibration at 25% B for 3 min at 350 µL/min and 0.5 min at 200 µL/min. The injection volume was 5 µL. The MS was operated in negative ionization mode with an ionization voltage of − 3000 V and a transfer tube temperature of 350 °C. The full scan experiments were conducted with a resolution of 60,000 FWHM and a range of *m*/*z* 100–800. The MS/MS experiments were conducted in parallel reaction monitoring mode with a resolution of 60,000 FWHM. The normalized collision energy was 30% for all analytes, except for *m*/*z* 353, for which it was 20%. The precursor ions were isolated at a window width of 1 m/*z* unit. Nitrogen was used as collision gas and generated by a CMC nitrogen generator (Eschborn, Germany). The MS was regularly calibrated using the factory-provided calibration solution.

### Assay characterization

The analytical method was validated for the qualitative detection of LGD-4033 in accordance with the WADA criteria for a confirmation procedure of a non-threshold substance. Additional assay parameters were characterized where deemed necessary for the estimation of concentration levels of LGD-4033. All validation samples were prepared following the SPE after enzymatic hydrolysis scheme (H-SPE) as described above. As ISTD, 10 µL of a working solution of S-24 (10 ng/mL in ACN) was added to all samples.

The selectivity of the method was determined by analyzing 10 blank urine samples of varying specific gravity (SG) and pH values. Additionally, the identification capabilities of the method were confirmed by positively identifying LGD-4033 at 2 ng/mL in 10 spiked urine samples. Urine samples of male (*n* = 6) and female (*n* = 4) volunteers were used. The same 10 blank samples and ISTD-normalized peak area were used for all subsequent validation criteria, unless indicated otherwise. The precision of the method was determined by fortifying 2 mL of blank urine with either 2 ng/mL, 500 pg/mL, or 100 pg/mL of LGD-4033. For the determination of the intra-day precision, the relative standard deviation of 10 samples was calculated. For the determination of the inter-day precision, the same work-up was done on three separate days and the relative standard deviation of 30 samples was calculated. For the limit of detection (LOD) and limit of identification (LOI), 10 spiked urine samples were analyzed at different concentrations between 2 ng/mL and 1 pg/mL. Detection criteria were the presence of at least the two diagnostic ion transitions *m*/*z* 337 to *m*/*z* 267 and *m*/*z* 337 to *m*/*z* 239 at the respective retention time of the analyte. Allowing for mass errors of 10 ppm, no background noise was observed in the extracted ion chromatograms of the selected ion transitions, and hence, the presence (or absence) of signals was confirmed visually. Identification criteria were the maximum tolerance window of relative abundances according to the WADA TD2021 IDCR for the three diagnostic ions *m*/*z* 267, *m*/*z* 239, and *m*/*z* 170 [23]. A sigmoidal fit function was constructed with the data, as explained in the WADA laboratory technical note on analytical method validation for doping control. The LOD and LOI were determined as the value of the sigmoidal fit function at 95% detection and identification rate respectively. Carryover of the analyte was determined by analyzing an extracted blank sample directly after the injection of a spiked urine sample at 8 ng/mL. For the determination of matrix effects, 10 blank urine samples were prepared with H-SPE and subsequently spiked with 4 ng LGD-4033, which is equivalent to 2 ng/mL LGD4033 in urine. The peak areas of the samples were compared to those of neat solvent spiked with the same amount of LGD-4033. Recovery was determined by comparing 10 spiked urine samples at 2 ng/mL in which the ISTD was added before the evaporation step to 10 urine samples where 2 ng/mL LGD-4033 and the ISTD were added before the evaporation step. The robustness of the chromatography was determined by calculating the relative standard deviation of the retention time of 10 spiked urine samples at 2 ng/mL. For the determination of linearity, a calibration curve with concentrations between 0.1 and 10 ng/mL was prepared and measured 6 times. The curves consisted of 11 calibration points. The extract stability was determined by re-measuring 10 spiked urine samples (2 ng/mL) after storage at + 8 °C for 7 days.

### Data evaluation and processing

The LC–MS/MS data were evaluated using Trace Finder 4.0 software by Thermo Fisher Scientific (Bremen, Germany). As there were no reference materials available for the metabolites, only relative intensities can be shown. The peak areas of the target ions were corrected with the peak area of the ISTD S-24. Additionally, all concentrations/peak areas of analytes were adjusted using the SG of the sample with the equation below. SG was measured with an ORF 1PM digital refractometer from Kern optics (Balingen, Germany).

$${\mathrm{Conc}}_{\mathrm{adjusted}}=\frac{(1.020-1)}{{\mathrm{SG}}_{\mathrm{sample}}-1}\bullet {\mathrm{Conc}}_{\mathrm{measured}}$$

The concentrations for LGD-4033 were estimated using a calibration curve between 0.1 and 10 ng/mL. To better estimate concentrations below 0.1 ng/mL, the calibration curves were forced through zero. For the visualization, average concentrations or intensity values for LGD-4033 and its metabolites, respectively, were determined for the shown excretion profiles. As not every sample was collected at the same time, the samples were combined in time slots to best represent all values. The numbers of data points per time point are visualized in the graphs, as a total of five data points were not available for every time point. Error bars were added for both time and intensity, indicating maximum and minimum values. Some data points were not included, if only one volunteer provided a sample at that time. A small amount of samples showed unusual matrix effects, which lead to suppression of the IS. These samples were diluted with water and the values corrected accordingly. As carryover was observed for the bis-hydroxylated metabolites M5-a and M5-b, the samples were measured in reverse order to minimize this effect. As a negative control sample was analyzed before each batch, a systematic contamination with analytes can be ruled out. With each batch, a calibration curve, consisting of at least 6 points, was prepared and measured. Statistical analysis was carried out with the data analysis tool in Excel from Microsoft (Redmond, WA, USA). Additional results from the application of 10 mg of LGD-4033 were published previously [18].

## Results and discussion

### Assay characterization

The LC–MS/MS method used for the qualitative analysis of LGD-4033 after H-SPE was comprehensively characterized and the results are listed in Table 1. The method was found to be selective and showed good inter- and intra-day precision at different concentrations. LOD and LOI were determined at 8 pg/mL and 125 pg/mL respectively. Recovery, ion suppression, stability, carryover, and robustness showed acceptable values and were deemed adequate for this application. The method is highly linear with *R*^2^-values of > 0.999.

**Table 1:** Validation results for the qualitative detection of LGD-4033

Intra-day precision	5.6%	at 2 ng/mL	*n* = 10
Intra-day precision	5.2%	at 0.5 ng/mL	*n* = 10
Intra-day precision	5.4%	at 0.1 ng/mL	*n* = 10
Inter-day precision	6.7%	at 2 ng/mL	*n* = 30
Inter-day precision	5.8%	at 0.5 ng/mL	*n* = 30
Inter-day precision	7.1%	at 0.1 ng/mL	*n* = 30
LOD	8	pg/mL	*n* = 10
LOI	125	pg/mL	*n* = 10
Selectivity	✓		*n* = 10
Recovery	73.8–100.0%	at 2 ng/mL	*n* = 10
Matrix effect	75.2–87.8%	at 2 ng/mL	*n* = 10
Extract stability (after 7 days)	92.9–101.6%	at 2 ng/mL	*n* = 10
Carryover	0.0%	at 8 ng/mL	*n* = 1
Robustness (CV RT)	0.06%	at 2 ng/mL	*n* = 10
Linearity (*R*^*2*^)	0.9998	0.1–10 ng/mL	*n* = 6

### Elimination studies

The collected urine samples were prepared with the developed H-SPE method. The SPE showed better extraction capabilities in comparison to liquid–liquid extraction, especially for the detection of the highly polar metabolites such as M5 and M6 (data not shown). The preceding hydrolysis with β-glucuronidase was added to ensure the maximum detection time of all metabolites, as previous studies demonstrated no increased detection times of glucurono-conjugated phase II metabolites [18]. As only phase I metabolites were included in this study, hereinafter, all mentioned metabolites are phase I metabolites.

A total of 6 metabolic pathways led to 15 metabolites in addition to the compound LGD-4033, which could be detected in excreted urine samples after the application of micro-doses of the SARM. For all described metabolites, mass spectrometric data has already been published prior to this study [15–17, 21]. The previously described increase of peak intensity and detection times by measuring the formate adducts could not be observed with this method [18]. Therefore, all analytes were detected using the deprotonated molecules [M-H]^−^ as precursor ions. A previously described methoxylated metabolite was not detected with this method [18].

The detected metabolites included the epimer of LGD-4033 (M1), hydroxylated and oxidized LGD-4033 (M2), mono-hydroxylated LGD-4033 (M3), LGD-4033 after hydroxylation and ring opening of the pyrrolidine ring (M4), bis-hydroxylated LGD-4033 (M5), and tri-hydroxylated LGD-4033 (M6). The chromatographic separation of the metabolites showed between two and four isomers with different retention times (see Table 2). These might be stereo- or regioisomers, of which some have been structurally elucidated in the literature [15, 16, 21]. To facilitate the estimation of concentrations of the metabolites, the synthesis of reference material will be necessary.

**Table 2:** All detected metabolites of LGD-4033

Analyte	Metabolic transformation	Exact mass [*m/z*]	Formula	Retention time [min]	Product ions [*m/z*]
LGD-4033	-	377.0781	C_14_H_11_F_6_N_2_O^−^	9.76	**267.0751** 239.0438 170.0223
M1	Epimerization			9.96	**267.0751** 239.0438 170.0223
M2-a	Hydroxylation, dehydrogenation	351.0574	C_14_H_9_F_6_N_2_O_2_^−^	7.19	**237.0645** 281.0543 253.0230
M2-b				7.40	**237.0645** 281.0543 253.0230
M2-c				8.00	**237.0645** 281.0543 253.0230
M2-d				8.40	**237.0645** 281.0543
M3-a	Hydroxylation	353.0730	C_14_H_11_F_6_N_2_O_2_^−^	8.55	**255.0751** 199.0489 185.0332
M3-b				8.82	**255.0751** 185.0332
M4-a	Hydroxylation, ring cleavage	355.0887	C_14_H_13_F_6_N_2_O_2_^−^	7.54	**285.0856** 257.0907 185.0332
M4-b				7.74	**285.0856** 257.0907 185.0332
M4-c				8.24	**285.0856** 185.0332
M4-d				8.40	**285.0856** 185.0332
M5-a	bis-Hydroxylation	369.0679	C_14_H_11_F_6_N_2_O_3_^−^	7.20	**281.0543** 237.0645 253.0594
M5-b				7.42	**281.0543** 237.0645 255.0751
M6-a	tri-Hydroxylation	385.0628	C_14_H_11_F_6_N_2_O_4_^−^	4.93	**185.0332** 269.0543
M6-b				6.03	**227.0438** 225.0645
S-24 (ISTD)		381.0868	C_18_H_13_O_3_N_2_F_4_^−^	9.70	**241.0594**

Product ions that are used for estimation of LGD-4033 or the determination of relative abundance of metabolites are marked in bold

**Fig. 1:**
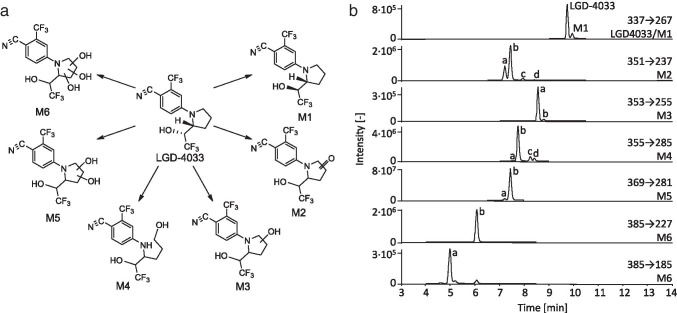
**a** Structures of LGD-4033 and main metabolites, and **b** extracted ion chromatograms of LGD-4033 and selected metabolites in a sample after the ingestion of five doses of 50 µg of LGD-4033. The sample was taken 8 h after the last ingestion of LGD-4033. The most intense product ion of each analyte is shown

All detected metabolites and their diagnostic ions used for the evaluation are listed in Table 2. The most intense product ions are indicated in bold and were chosen as target ions. Depending on the analyte, either one or two additional confirming ions were chosen. Extracted ion chromatograms of the detected analytes are shown in Fig. 1. The metabolites M2-a and M2-b elute with a very similar retention time as M5-a and M5-b. It could not yet be determined if M2-a and M2-b are true metabolites as postulated by Geldof et al. or in-source fragments of M5-a and M5-b as postulated by Cox et al. [15, 21].

### Single-dose studies

**Fig. 2:**
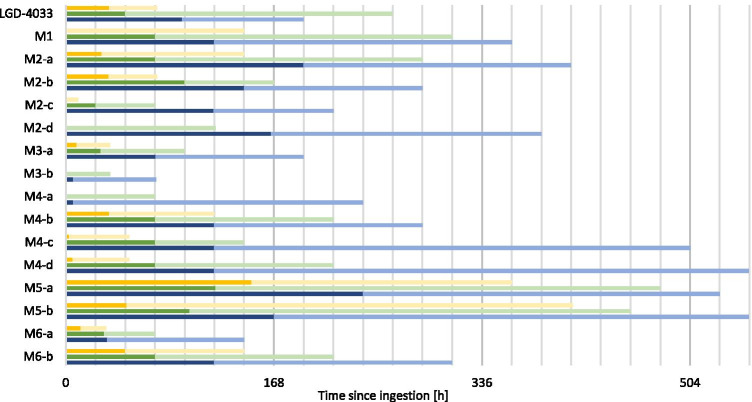
Detection times of LGD-4033 and its metabolites after the ingestion of one dose of 1 µg (yellow), 10 µg (green), or 50 µg (blue) of LGD-4033. The minimum detection times are shown in dark color and maximum detection times are depicted in lighter color. For each dose, the detection times of five volunteers were used

Even at the low administered doses, LGD-4033 and its metabolites can be detected with the developed method for multiple days or weeks depending on the analyte. The detection times of the analytes are shown in Fig. 2. The minimum and maximum detection times of the analytes varied notably between the volunteers. M2-d, M3-b, and M4-a could not be detected in any sample after 1 µg application. M1 and M2-c could only be detected in some volunteers after 1 µg application. M2-d, M3-b, and M4-a could only be detected in some volunteers after 10 µg application. All analytes could be detected in all volunteers after 50-µg application. The analyte that shows the longest detection window is M5-a, with the earliest negative sample after 11 days (50 µg). Therefore, this metabolite is the ideal target for doping analysis. The maximum concentrations of LGD-4033 that were detected after single-dose application were 2.5–4.0 ng/mL (50 µg), 0.4–1.1 ng/mL (10 µg), and 0.05–0.1 ng/mL (1 µg). These maximum values were detected between 2 and 4 h after application in all studies. It can therefore be concluded that although the detection times of LGD-4033 and its metabolites vary notably between individuals, the excretion behavior and metabolism are quite similar in the first hours after application.

**Fig. 3:**
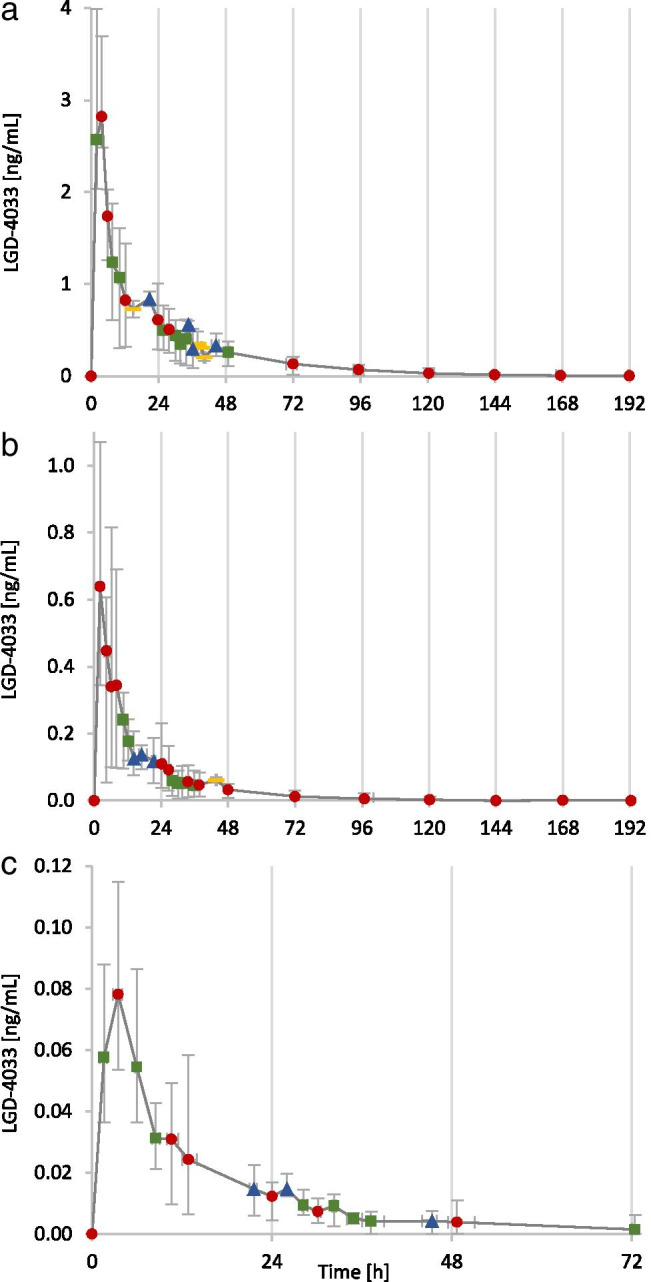
Elimination profiles after the single ingestion of **a** 50 µg, **b** 10 µg, and **c** 1 µg of LGD-4033. Shown are the averages of multiple data points with error bars to indicate maximum and minimum values. The number of underlying data points is shown as red circle (5), green square (4), blue triangle (3), and yellow bar (2)

In Fig. 3, the elimination profiles of LGD-4033 after the intake of 1, 10, and 50 µg LGD-4033 are shown. A pilot study with one volunteer showed that after the ingestion of 10 ng of LGD-4033, the metabolite M5-b is detected for 72 h (data not shown).

### Multi-dose studies

**Fig. 4:**
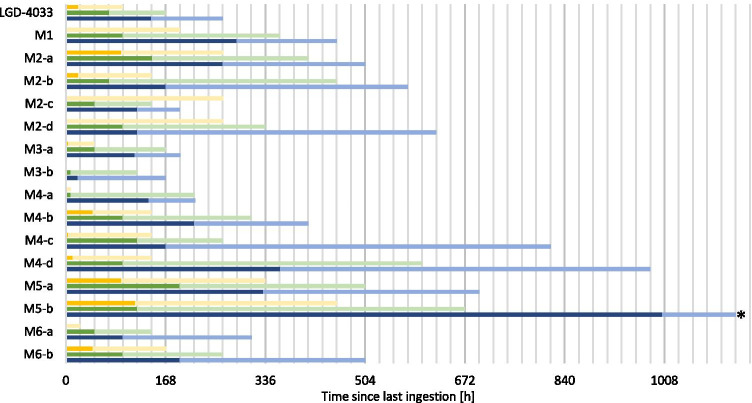
Detection times of LGD-4033 and its metabolites after the ingestion of five doses of 1 µg (yellow), 10 µg (green), or 50 µg (blue) of LGD-4033 in 5 days. The detection times are calculated after the last ingestion of LGD-4033 on the fifth day. The minimum detection times are shown in dark color and maximum detection times are depicted in lighter color. For each dose, the detection times of five volunteers were used. *As no samples were collected after 1128 h after last ingestion, no definitive maximum detection time can be determined

In addition to the single-dose studies, multi-dose studies were performed to simulate the daily intake of LGD-4033 and investigate its potential influence on metabolite ratios and accumulation behavior. In Fig. 4, the minimum and maximum detection times of LGD-4033 and its metabolites are shown. M3-b and M4-a could not be detected in any sample after multiple 1 µg applications. M1, M2-c, M2-d, and M6-a could only be detected in some volunteers after multiple 1 µg applications. All analytes included in this study were detected in all volunteers’ urine samples after multiple 10 µg and 50 µg applications. For M5-b, no maximum detection time for the multiple 50 µg applications can be determined because M5-b was detected in all samples until the end of the study 1004 h and 1128 h after the last ingestion of LGD-4033.

**Fig. 5:**
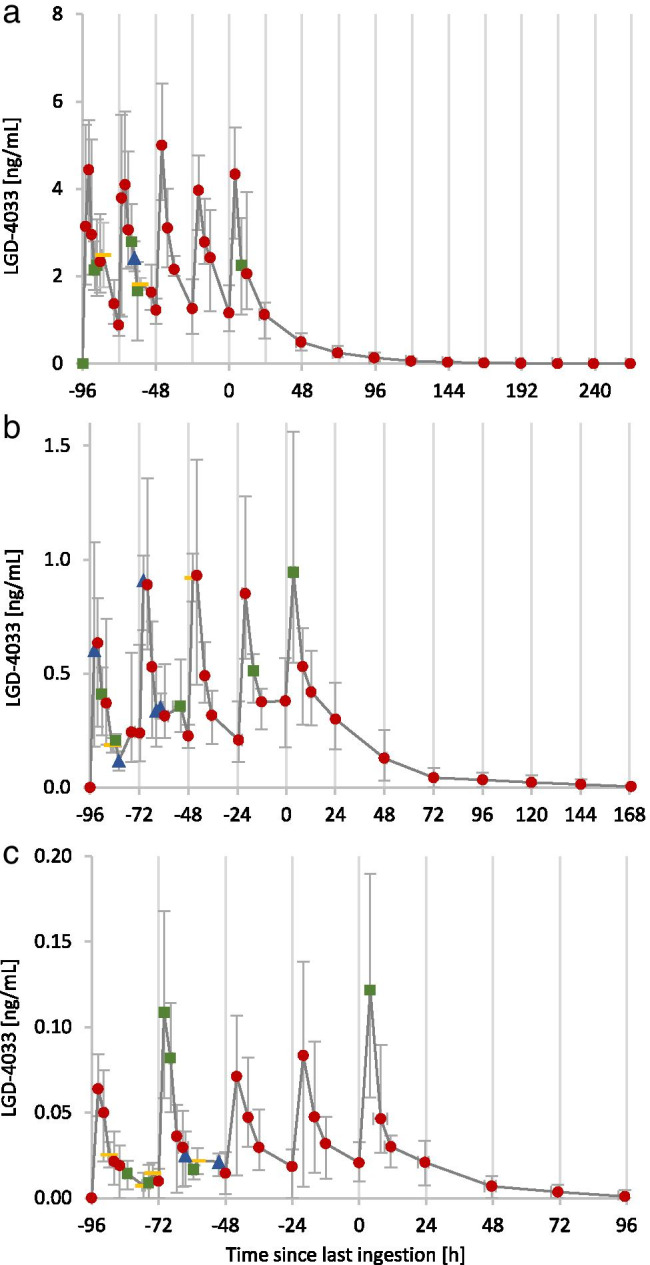
Elimination profiles after the five ingestions of **a** 50 µg, **b** 10 µg, and **c** 1 µg of LGD-4033. Shown are the averages of multiple data points with error bars to indicate maximum and minimum values. The number of underlying data points is shown as red circle (5), green square (4), blue triangle (3), and yellow bar (2)

In Fig. 5, the elimination profiles of LGD-4033 after the intake of five doses of 1, 10, and 50 µg LGD-4033 are shown. Five distinct maxima could be detected after each ingestion with *t*_max_ of 2–5 h (50 µg), 2–8 h (10 µg), and 2–13 h (1 µg). No considerable accumulation of LGD-4033 after multiple ingestions of micro-doses can be observed. The metabolites M1, M2a, M2d, M3b, M4c, M4d, and M5a show some accumulation after multiple doses (data not shown). The maximum concentrations of LGD-4033 that were detected after multi-dose application were 4.3–6.4 ng/mL (50 µg), 0.7–1.6 ng/mL (10 µg), and 0.05–0.2 ng/mL (1 µg). As with the single-dose application, the inter-personal variability between volunteers is high.

### Metabolite ratios

A particularity of the metabolism of LGD-4033 is the relative metabolite ratio of isomers that changes over time. As most metabolites are excreted in more than one isomeric form (see Table 2), the ratio between the different isomers can be used to estimate a possible time span since ingestion of LGD-4033. The main goal of this study is to contribute analytical data for best-possible result management in support of differentiating a recent intake of (or exposure to) low amounts of LGD-4033 as opposed to a tail end excretion of a pharmacologically relevant amount of the drug candidate. As the mere concentration of LGD-4033 in a sample does not provide sufficient information, the combined metabolite ratio and LGD-4033 concentration determined in doping control urine samples can be compared to data shown in this study to probe for scenarios compatible with micro-doses. The analyte pair that shows the most promising results is LGD-4033 and its epimer M1. Although M5-a and M5-b also show a ratio shift over time and offer the longest detection times, the ratio of M5-a and M5-b could not be employed for this approach as its increase is followed by a decline at a later stage of drug elimination, which prevents a reliable interpretation of the time line (data not shown).

M1 was synthesized by oxidation of the alcohol moiety of LGD-4033 utilizing a Dess-Martin reaction and subsequently reducing the resulting carbonyl moiety. This yielded a mixture of LGD-4033 and M1, which was separated on a preparative LC (see Methods—“Synthesis of M1”). The structure of M1 was confirmed as identical to LGD-4033 by ^1^H-NMR spectroscopy and the shift in retention time of 0.2 min in the LC–MS/MS method supports the identity of M1 as the epimer of LGD-4033.

**Fig. 6:**
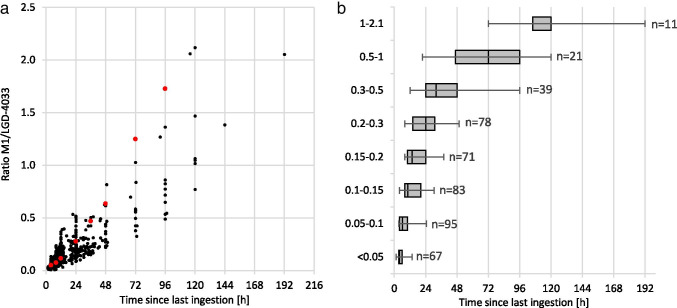
Combined intensity ratios of M1 and LGD-4033 as a function of time passed since ingestion as **a** in black a scatterplot of all micro-dose data points and in red the data after 10 mg application [18] and **b** boxplots created with all data points. The box represents 50% of data points in each ratio range, and the whiskers represent the top and bottom 25% of data points. The median is visualized by a black line within the box. The number of underlying data points is shown next to each boxplot

The ratio M1/LGD-4033 is shown in Fig. 6a for all excretion study samples in which LGD-4033 could be positively identified using the mass spectrometric identification criteria (see Methods—“Assay characterization”). This approach reduces outliers, as the ratio tends to be unstable at low concentrations. To better simulate the likely contamination scenario of the daily intake of small amounts of LGD-4033, the ratio values of the multi-dose study were also included in the underlying data. With each new ingestion, the time was set back to 0 h. As some volunteers showed slightly increased ratios after multiple applications, the values between 0 and 4 h after a repeated ingestion were not included in the data to minimize this effect. The resulting data was tested for linear correlation with a resulting Pearson’s correlation coefficient of 0.87, a coefficient of determination of 0.76, and a *p*-value of < 0.001. The slope was determined at 0.0091 and the *y*-intercept at 0.018.

In addition to the micro-dose data, one set of previously collected excretion study urine samples collected after the ingestion of 10 mg of LGD-4033 was re-analyzed with the developed method [18]. The M1/LGD-4033 ratios of these samples are depicted in red in Fig. 6a. These data points lie within the limits estimated from the micro-dose data, suggesting that the M1/LGD-4033 is dose-independent, but more controlled excretion studies would be necessary to corroborate that claim.

The data points were combined into ratio ranges and visualized using boxplots (Fig. 6b). The highest and lowest values in each range were set as limits for the estimation of the time passed since the ingestion of LGD-4033. Limitation of this approach is the inter-individual variation of the ratio/time relationship, which appears to increase with the time elapsed since ingestion. The limits were devised with the data of 30 excretion studies conducted with 13 different volunteers. Due to the low number of data points for M1/LGD-4033 ratios > 0.5, the informative value of the approach is reduced.

In addition to the time passed since the ingestion, the dose that was ingested can be roughly estimated by comparing the determined urinary LGD-4033 concentration to the range of concentrations measured in the micro-dose studies using the time range estimated from the M1/LGD-4033 abundance ratio. As the time ranges are large and the urinary concentrations of the target analytes vary between individuals, a substantial error of this approach cannot be excluded. Limitations of this approach include the small sample size of 30 studies conducted with 13 male volunteers. Age, weight, and athletic ability were not considered in the evaluation of the data. Nonetheless, this data is an important first step in estimating time and dose of LGD-4033 intake, contributing to decision-making processes in cases of AAFs.

### Example of time and dose estimation

An exemplary scenario of the application of the method is presented here. A urinary concentration of 0.2 ng/mL of LGD-4033 with a M1/LGD-4033 ratio of 0.12 is detected in a routine doping control sample of an athlete. The question whether the athlete unknowingly ingested LGD-4033 through a daily consumed DS is raised, and analyses of the product reveal a contamination that results in a daily intake of approximately 10 µg of LGD-4033. Using the measured M1/LGD-4033 ratio and the correlation shown in Fig. 6, the intake of LGD-4033 is estimated to have occurred between 4 and 30.5 h before urine sample collection. Further, to estimate the dose of LGD-4033 that was ingested, the concentration of the sample is compared to the concentration range detected during the excretion studies. The concentrations of LGD-4033 in post-administration urine samples (10 µg of LGD-4033) collected between 4 and 30.5 h are found between 0.006 and 0.8 ng/mL, hence supporting the scenario of a DS being the source of the SARM ingested by the athlete.

### Conclusion

As the analytical capabilities in doping control laboratories have improved in the past years, the retrospectivity and thus the necessity to differentiate between deliberate doping and unintentional exposure to prohibited substances have substantially increased. Here, human micro-dose elimination studies can provide critical data for decision-making processes in anti-doping. In this study, the elimination behavior of LGD-4033 after oral application of microgram amounts was examined by performing controlled elimination studies and analyzing collected urine samples with a developed LC–MS/MS method, which was validated for the qualitative detection of LGD-4033 in accordance with the WADA criteria. In addition to LGD-4033 itself, 15 metabolites were detected in human urine. Information on the elimination behavior, as well as the detection times of the different metabolites, was generated. The abundance ratio of the isomeric analytes LGD-4033 and M1 was shown to exhibit a linear relationship with the time passed since the ingestion. Therefore, using the analyte ratio in an unknown sample, an estimation about the time point of drug exposure/administration is possible. By comparing the urinary LGD-4033 concentration of the unknown sample to the concentration range of the micro-dose data, a rough estimation of the administered dose can also be made. The limitation of this approach is the low number of samples providing the data that compose the interpretation scheme. For more robust information, additional elimination studies with a larger number of volunteers and further dosing (and alternative routes of administration) appear warranted.

## Data Availability

The datasets generated and analyzed during the current study are available from the corresponding author on reasonable request.
